# Impact of lifestyle interventions on reproductive and psychological outcomes in women with polycystic ovary syndrome: A systematic review

**DOI:** 10.1097/MD.0000000000041178

**Published:** 2025-01-17

**Authors:** Amal H. Mohamed, Osama Albasheer, Manar Ahmed Ghoniem, Nagla Abdalghani, Fatma Ayish, Siddig Ibrahim Abdelwahab, Maha Murtada Abdelmageed, Ahlam Mohammed S. Hakami, Ali Hassan Khormi, Ahmed Abdallah Altraifi, Isameldin Medani, Uma Chourasia, Suhaila A. Ali, Amani Abdelmola, Anas E. Ahmed

**Affiliations:** a Department of Internal Medicine, Faculty of Medicine, Jazan University, Jazan, Saudi Arabia; b Department of Family and Community Medicine, Faculty of Medicine, Jazan University, Jazan, Saudi Arabia; c Internal Medicine, Respiratory Therapy Department, Faculty of Applied Medical Sciences, Jazan University, Jazan, Saudi Arabia; d Internal Medicine, Internal Medicine Department, Faculty of Medicine, Jazan University, Jazan, Saudi Arabia; e Health Research Centre, Jazan University, Jazan, Saudi Arabia; f Department of Obstetrics and Gynecology, Obstetrics and Gynecology, Faculty of Medicine, Jazan University, Jazan, Saudi Arabia.

**Keywords:** dietary, exercise, intervention, obesity, quality of life, reproductive health

## Abstract

**Background:**

Polycystic ovary syndrome (PCOS) is a complex endocrine disorder affecting ≈8% to 13% of women of reproductive age. PCOS has multifaceted effects that extend beyond reproductive health. Women with PCOS are at an elevated risk for various metabolic conditions, including obesity, type 2 diabetes, and cardiovascular disease, as well as psychological challenges, such as anxiety, depression, and reduced quality of life. This systematic review examined the effectiveness of lifestyle interventions, including dietary, exercise, and behavioral modifications, in improving reproductive outcomes, mental well-being, and quality of life in women with PCOS.

**Methods:**

A comprehensive search was conducted using MEDLINE, EMBASE, Cochrane Library, and Web of Science databases, identifying observational and interventional studies published in English through December 2022. Studies were evaluated for methodological quality and categorized according to the type of lifestyle intervention and outcome measures.

**Results:**

Of the 24 studies reviewed, with 16 focusing on reproductive outcomes, 4 on quality of life, and 4 on combined outcomes, encompassing 1373 participants with the mean age in the included studies ranged from 21.7 to 36.5 years. Dietary modifications, either alone or in combination with exercise, resulted in significant improvements in reproductive health, including a 5% reduction in body weight (*P* < .001), increased menstrual regularity, higher pregnancy rates, and decreased testosterone levels (*P* < .01). Exercise interventions further contributed to positive outcomes; a 20-week exercise program improved ovulation rates by 49.1% and significantly reduced testosterone and free androgen indices (*P* < .001). In addition, structured exercise programs increased menstrual regularity by 60% and reduced body mass index and testosterone levels.

**Conclusion:**

This review underscores the efficacy of integrated lifestyle interventions, including dietary, exercise, and behavioral approaches, in improving reproductive health, psychological well-being, and quality of life in women with PCOS. These findings highlight the potential of comprehensive nonpharmacological management strategies to address the multifaceted health challenges posed by PCOS. Future research should prioritize long-term studies to assess sustained outcomes and examine personalized intervention strategies that account for the clinical diversity and heterogeneity of PCOS presentations.

## 1. Introduction

Polycystic ovary syndrome (PCOS) is a prevalent endocrine and reproductive disorder characterized by hyperandrogenism, polycystic ovarian morphology, and ovulatory dysfunction, affecting an estimated 6% to 10% of women of reproductive age.^[1]^ This condition is frequently accompanied by a spectrum of metabolic disturbances, such as insulin resistance, obesity, dyslipidemia, and a heightened risk of cardiovascular disease, which collectively amplify its health impact.^[2–5]^ In addition, PCOS is frequently accompanied by a profound impact on self-esteem, body image, and overall mental well-being.^[3]^ While the precise pathogenesis of PCOS remains incompletely understood, genetic factors, disruptions in adipokines, proteins secreted by adipose tissue with roles in metabolic regulation, and inflammatory mediators are emerging as significant contributors to its development and progression.^[6,7]^

The complexity of PCOS, marked by its metabolic, reproductive, and psychological manifestations, necessitates a comprehensive management strategy.^[8,9]^ Lifestyle interventions, including dietary, exercise, and behavioral modifications, are increasingly recognized as foundational components in the management of PCOS. These nonpharmacological approaches have shown potential benefits in improving reproductive health, insulin sensitivity, weight control, and overall quality of life.^[10–13]^ However, despite promising findings, the evidence remains varied regarding the effectiveness of specific intervention types and combinations, particularly in terms of long-term outcomes.

A gap persists in synthesizing robust, comparative evidence on how lifestyle interventions, whether as standalone or combined approaches, address the diverse challenges of PCOS. This systematic review aimed to critically evaluate the current literature on dietary, exercise, and behavioral interventions in PCOS, with a focus on reproductive, psychological, and quality-of-life outcomes, to provide a clearer understanding of their effectiveness and inform future research directions.

## 2. Materials and methods

### 2.1. Study design

This systematic review was conducted in accordance with the Preferred Reporting Items for Systematic Reviews and Meta-Analyses guidelines^[14]^ to ensure transparency and rigor in the review process. The population, intervention, comparator, outcomes, and study design framework were used to define the eligibility criteria and guide the selection of studies. All steps, including study selection, data extraction, and bias assessment, were documented and independently verified by the reviewers.

### 2.2. Eligibility criteria

Studies were considered eligible for inclusion if they examined women of reproductive age diagnosed with PCOS, as defined by standardized criteria, such as the Rotterdam, National Institutes of Health (NIH), or Androgen Excess and PCOS society criteria. This review focused on studies evaluating the impact of lifestyle interventions, including dietary modifications, exercise programs, and behavioral therapy, on outcomes related to reproductive health, psychological well-being, and quality of life. Observational and interventional study designs, such as randomized controlled trials (RCTs), nonrandomized trials, and cohort studies, were included to provide a comprehensive perspective.

To ensure the relevance and reliability of the data, only studies published in English with quantifiable outcome measures were included. Articles that lacked full-text availability, focused solely on pharmacological or surgical treatments, or employed nonstandard diagnostic criteria for PCOS were excluded. In addition, systematic reviews, ongoing trials, and studies with insufficient methodological details or a significant risk of bias were omitted.

### 2.3. Search strategy

A comprehensive search was conducted across multiple databases, including MEDLINE, EMBASE, Cochrane Library, and Web of Science, for studies published up to December 2022. Keywords and medical subject heading terms related to “PCOS,” lifestyle interventions, “diet,” exercise, “behavioral therapy,” reproductive health, “psychological health,” and “quality of life” were used. Reference lists of the included studies and gray literature were manually searched to identify additional relevant articles.

### 2.4. Study selection

Two independent reviewers screened the titles and abstracts to identify eligible studies. Full-text articles were reviewed based on inclusion and exclusion criteria. Any discrepancies between the reviewers were resolved through discussion or consultation with a third reviewer.

### 2.5. Assessment of bias

The risk of bias within each included study was assessed using the Cochrane Risk of Bias 2 tool.^[15]^ This framework focuses on 5 domains: bias arising from the randomization process, deviations from intended interventions, missing outcome data, outcome measurement, and selection of the reported result. Each domain was rated as “low risk,” “some concerns,” or “high risk” based on signaling questions, and an overall risk of bias rating was assigned to each study.

To further minimize bias in the systematic review process, we addressed publication bias by conducting an extensive search of gray literature and manually reviewing reference lists to capture relevant unpublished studies. Selection bias at the review level was mitigated by following predefined inclusion criteria, ensuring a systematic and comprehensive approach for study selection. Two independent reviewers conducted all assessments and discrepancies were resolved through discussion or consultation with a third reviewer to enhance consistency and reliability.

### 2.6. Data extraction and synthesis

Data extraction was performed independently by 2 reviewers and included study characteristics (author, year, and design), participant demographics (sample size, age, and body mass index [BMI]), intervention details, outcomes assessed, and key findings. The extracted data were tabulated and qualitatively synthesized to highlight patterns in outcomes across dietary, exercise, and behavioral interventions.

### 2.7. Handling heterogeneity

Owing to the heterogeneity in study designs, interventions, and outcomes, a narrative synthesis approach was employed. This allowed for the integration of findings across diverse methodologies while accounting for variations in study populations and intervention protocols.

### 2.8. Ethical considerations

As this study involved a review of published literature, no ethical approval was required.

## 3. Results

### 3.1. Search results

**Figure 1.**
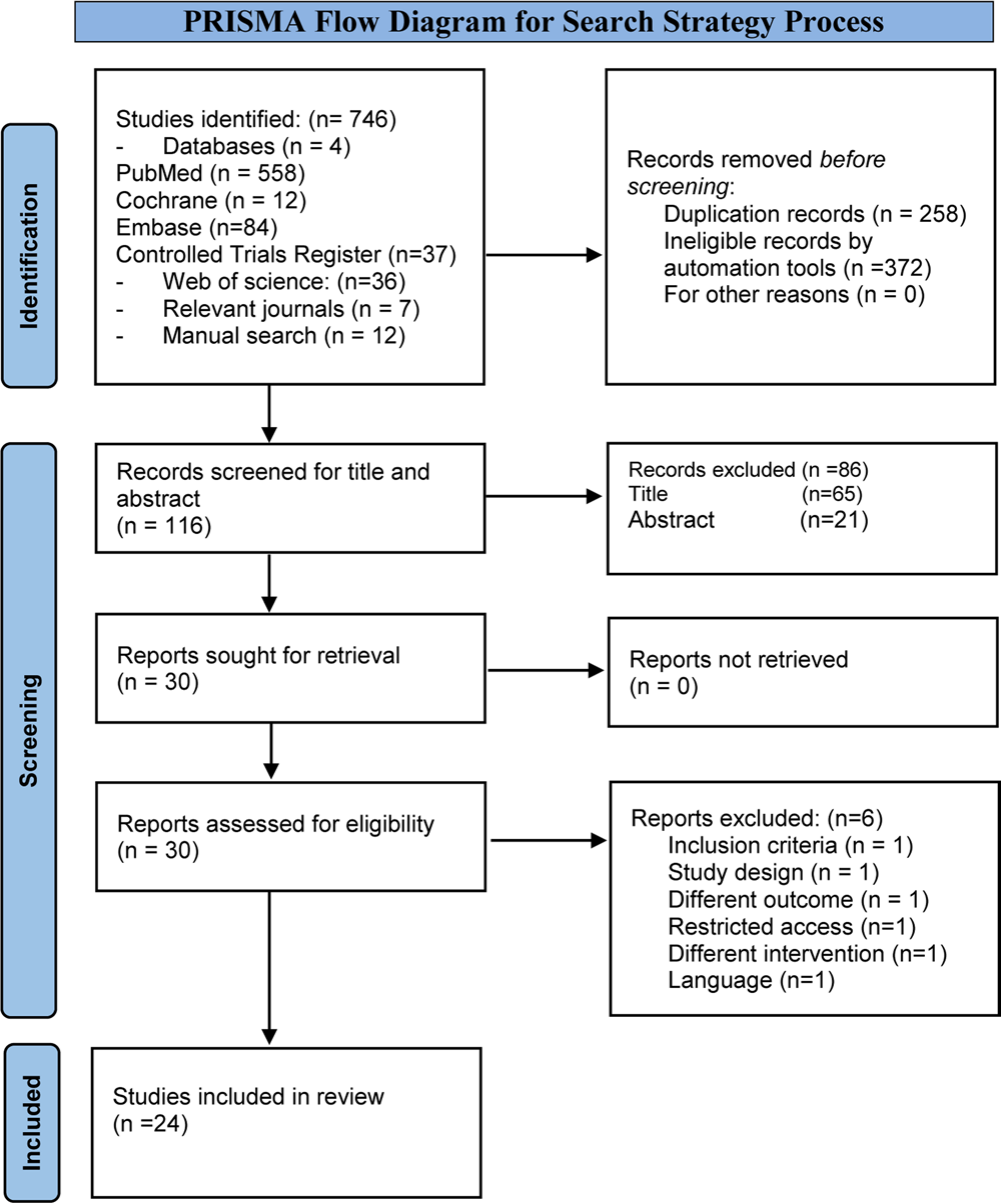
Preferred Reporting Items for Systematic Reviews and Meta-Analyses (PRISMA) flow diagrams for search strategy process.

The sequence of the identification and screening processes is displayed in the Preferred Reporting Items for Systematic Reviews and Meta-Analyses flow diagram (Fig. 1); a total of 764 publications were retrieved. Records were identified from 4 main databases: PubMed (n = 558), Cochrane (n = 12), Controlled Trials Register (n = 37), and EMBASE (n = 84). Other methods and sources for the identification of studies were the Web of Science (n = 36) relevant journals such as the Lancet and the New England Journal of Medicine (n = 7). More articles were found by manually searching and looking up references from the identified studies (n = 12).

The Rayyan online tool^[16]^ was used to assess duplication, and (n = 258) duplicate articles were excluded. For the remaining identified research (n = 488 articles), an automated filter for the database, by using filter boxes such as study design (for RCTs, clinical trials, and observational studies) and age group, was used to narrow the search to relevant articles that met the inclusion criteria, by which a total of 372 articles were excluded. The title and abstract were selected and evaluated (n = 116), and, accordingly, another 86 articles were eliminated (n = 65) by title and (n = 21) by abstract evaluation.

After excluding publications based on the title or abstract, 30 full-text articles were extracted for a more thorough analysis. Following full-text screening, another 6 articles were excluded because they did not meet the eligibility criteria. The remaining 24 articles were deemed eligible for inclusion, and their methodological quality was assessed with the results reported in this review.

### 3.2. Characteristics of the included studies

In this systematic review, a total of 24 articles composed of 1373 participants, which were found to be relevant to the review question and meeting the inclusion criteria, were evaluated (Table 1) with a mean age of the participants, in the included studies, ranging between 21.7 ± 2.3 and 36.5 years. Four studies^[17–20]^ displayed age as a range rather than a mean, while only 1 study did not state age.^[21]^ As there is no time limitation, these articles were published from 1992 to 2022.

**Table 1:** Overview of the included studies.

Author	Study design	Sample size	Characteristics of the participants			Intervention	Outcome
			Age, y	BMI, kg/m²	Diagnosis		
Kiddy et al^[21]^	Within group comparison	24	Not stated	34.1 ± 4.9	Pelvic USG Clinical and biochemical hyperandrogism	Diet	Reproductive health
Moran et al^[30]^	RCT	45	33 ± 0.84	37.4 ± 1.24	Clinical and/or biochemical hyperandrogenism	Diet	Reproductive health
Stamets et al^[31]^	RCT (pilot)	35	Intervention: 29 ± 4 Control: 26 ± 4	Intervention: 38 ± 4 Control: 37 ± 5	Clinical and unexplained elevated circulating T levels	Diet	Reproductive health
Bruner et al^[41]^	RCT (pilot)	12	28.4 ± 2.7 32.3 ± 1	36.6	Rotterdam criteria	Exercise + behavioral management	Reproductive health
Vigorito et al^[25]^	RCT	90	Intervention: 21.7 ± 2.3 Control: 21.9 ± 1.9	Intervention: 29.3 ± 2.9 Control: 29.4 ± 3.5	ESHRE/ASRM	Exercise	Reproductive health
Thomson et al^[26]^	RCT	94	29.3 ± 0.68	36.1 ± 4.8	Rotterdam criteria	Diet + exercise	Reproductive health
Palomba et al^[22]^	Non-RCT	40	Intervention: 26.8 ± 5.1 Control: 25.8 ± 4.5	Intervention: 33.1 ± 1.3 Control: 33.2 ± 1.4	Rotterdam ESHRE/ASRM/NIH	Exercise	Reproductive health
Kordi et al^[32]^	RCT	24	22.7 ± 3.77	27.53 ± 5.02	Trans-vaginal U/S	Exercise	Reproductive health
Nybacka et al^[17]^	RCT	57	18–40 yr	>27 kg/m^2^	Rotterdam consensus	Diet	Reproductive health
De Frène et al^[24]^	Prospective longitudinal within-patient study	31	29 yr	≥25 kg/m² 33.74	Rotterdam consensus	Diet + exercise + behavioral management	Quality of life
Turan et al^[35]^	RCT	32	24.45 ± 2.8 yr	Intervention: 14, 21.8 ± 1.0 Control: 16, 21.9 ± 1.1	ESHRE/ASRM	Exercise	Reproductive health
Almenning et al^[37]^	RCT (pilot)	31	27.2 ± 5.5 yr	26.7 ± 6.0	ESHRE/ASRM	Exercise	Reproductive health
Stefanaki et al^[40]^	RCT	23	Intervention: 23.4 ± 4.62 Control: 28.3 ± 7.20	Intervention: 21.53 ± 2.15 Control: 23.7 ± 4.4	Rotterdam definition	Behavioral management	Quality of life
Ramos et al^[27]^	Non-RCT case-control study	124	Intervention: 27.8 ± 5.34 Control: 29.74 ± 5.26	PCOS: 27.91 ± 5.51 Control: 25.99 ± 5.49	Rotterdam consensus	Exercise	Reproductive health Quality of life
Vizza et al^[33]^	RCT	15	27	37.8	Not mentioned	Exercise	Reproductive function, psychological, and quality of life
Deepthi et al^[19]^	Within group RCT	30	18–25 yr	26.11 kg/m^2^	Not mentioned	Exercise	Quality of life
Vasheghani-Farahani et al^[38]^	RCT	40	Control: 29 ± 5.39 Exercise: 27.7 ± 4.2	Control: 25.7 ± 2.15 Exercise: 28.8 ± 5.88	Rotterdam criteria	Exercise	Reproductive health
Mani et al^[28]^	RCT	161	33.4 ± 7.6	Control = 33.2 (6.2) Intervention = 34.2 (7.2)	ESHRE/ASRM	Behavioral management	Quality of life Reproductive health Psychological health
Costa et al^[20]^	RCT	27	18–34 yr	25 and 39.9 kg·m^−2^	Rotterdam criteria	Exercise	Quality of life
Oberg et al^[18]^	RCT	68	18–40 yr	≥27	Rotterdam PCOS criteria	Behavioral management	Reproductive health
Cochrane et al^[23]^	Quasi-experimental	15	Intervention: 30.1 ± 4.6 Control: 37.5 ± 4.0	>28.6	Not mentioned	Exercise + diet	Reproductive health
Liu et al^[29]^	RCT	296	Intervention: 31.79 ± 3.38 Control: 32.31 ± 3.79	Intervention: 27.50 ± 0.24 Control: 27.63 ± 0.28	Modified Rotterdam criteria	Diet + exercise	Reproductive health
Benham et al^[39]^	RCT	60	29.2 ± 4.7 yr	31.4 ± 8.4 kg/m^2^	Rotterdam criteria	Exercise	Reproductive health
D’souza et al^[34]^	RCT	30	Intervention: 24.26 ± 3.84 Control: 24.20 ± 3.54	23.39 ± 6.16 Not stated for control	Not mentioned	Diet + exercises + behavioral management	Reproductive health Quality of life

ASRM = American Society of Reproductive Medicine, BMI = body mass index, ESHRE = European Society of Human Reproduction and Embryology, NIH = National Institutes of Health, PCOS = polycystic ovary syndrome, RCT = randomized controlled trial, U/S = United States, USG = ultrasound sonography.

The studies varied considerably in their designs, populations, interventions, and reported outcomes, reflecting the complex and multifaceted nature of managing PCOS through lifestyle interventions. The included studies showed heterogeneity in several key areas. The study designs ranged from RCTs (19 studies), nonrandomized,^[22]^ and quasi-experimental studies,^[23]^ with varying levels of methodological rigor.^[19,21,24]^

Although most of the research had small to medium sample sizes ranging from 15 to 68 participants, on the other hand, 4 studies had larger sample sizes of 90, 94, 124, 161, and 296 women,^[25–29]^ respectively. PCOS was diagnosed according to the Rotterdam criteria in 11 articles; European Society of Human Reproduction and Embryology (ESHRE)/American Society of Reproductive Medicine (ASRM) was used to establish the diagnosis in 4 studies. Palomba et al^[22]^ used 3 criteria (Rotterdam ESHRE/ASRM/NIH, ASRM, and NIH). Clinical and biochemical hyperandrogenism of the pelvic ultrasound sonography were considered as diagnostic methods by Kiddy et al,^[21]^ Moran et al,^[30]^ Stamets et al,^[31]^ and Kordi et al.^[32]^ The criteria used for PCOS diagnosis were not specified in 4 studies.^[19,23,33,34]^

Participant populations were diverse, with some studies focusing exclusively on obese women, while others included participants with broader BMI ranges.

Two studies specifically evaluated nonoverweight and nonobese females.^[35,36]^ All studies were of duration that lasted >2 weeks.

The interventions varied widely, encompassing dietary modifications, exercise programs, behavioral strategies, or combinations of these approaches, with durations ranging from 8 weeks to 12 months.

### 3.3. Characteristics of the interventions, outcomes, and findings

The characteristics of the interventions, results, and findings are illustrated in Table 2, which includes research with only dietary intervention (n = 4).^[17,21,30,31]^ Dietary modifications with different compositions were used, such as low-calorie, low-fat diets as well as low- and high-protein diets. Despite these differences, however, certain trends and patterns have emerged. Dietary interventions are particularly effective in improving menstrual regularity, ovulation, and hormonal balance, with benefits closely tied to weight reduction and improved insulin sensitivity. A low-calorie diet with phased caloric restriction (330 kcal/d, followed by 1000 kcal/d) resulted in over 5% body weight loss (*P* < .001). This intervention improved menstruation and pregnancy rates, and significantly reduced testosterone levels (*P* < .01). Low-protein (55% carbohydrates) versus high-protein (40% carbohydrates) diets were tested for 16 weeks. Both led to improved menstrual regulation, pregnancy rates, and BMI reduction, with notable benefits in reproductive hormone profiles despite a dropout rate of 37.7%. A hypocaloric, high-protein diet achieved significant weight loss and improvements in reproductive function, including hormonal indices, within just 1 month.

**Table 2:** Characteristics of the interventions, outcomes, and findings.

Study	Duration	Description of the intervention	Outcome	Dropout rate	Findings
Kiddy et al^[21]^	7 mo	Low calorie: BMI > 30 kg/m^2^, 330 kcal/d for 4 wk, followed by a 1000 kcal/d for 6 mo, BMI = 25–30 kg/m^2^, 1000 kcal Low-fat diet, 20 g fat/d	Clinical and biochemical indices of reproductive function	0%	>5% loss of BW (*P* < .001), improvement in menstruation Pregnancy rate Reduction in testosterone (*P* < .01)
Moran et al^[30]^	16 wk	Low protein: 55% carbohydrate, 15% protein, and 30% fat High protein: 40% carbohydrate, 30% protein, and 30% fat	BW, menstrual regulation, ovulation, hirsutism, and reproductive hormone profile	37.7%	Improvement in menstruation Pregnancy rate Reduction of BMI
Stamets et al^[31]^	1 mo	High protein: 30% protein, 40% carbohydrate, and 30% fat or High carbohydrate:15% protein, 55% carbohydrate, and 30% fat	BW hormonal indices of reproductive function	25.7%	Significant weight loss and significant improvement in reproductive function in hypocaloric diet
Bruner et al^[41]^	12 wk	Endurance and resistance exercise plus nutritional counselling 3 d/wk. 10 min worm-up and 30 min exercise 1 hr/wk of nutritional counseling	Hormonal, menstrual, and reproductive function	0	Overall improvement in hormonal indices and menstruation
Vigorito et al^[25]^	3 mo	Training sessions 3 times/wk, 5-min warm up and cool down, and then 30-min exercise	Reproductive function; menses diary, and testosterone	0	60% normal menstrual cycle Significant improvement of BMI and testosterone
Thomson et al^[26]^	20 wk	Energy-restricted, high-protein diet, and/or walking/jogging program 5 sessions/wk Exercise intensity progressed from 25 to 30 min during the first week to 45 min by study end	Reproductive function	44.6%	Significant improvement in BW, testosterone, and FAI (*P* < .001) and ovulation (49.1%)
Palomba et al^[22]^	24 wk	Three training session on a bicycle ergometer/30 min/3 times/wk. High-protein composition and restricted caloric diet	Pregnancy rate, menstrual cycles, and fertility BMI	15%	Both groups improve fertility
Kordi et al^[32]^		Training session on a bicycle ergometer/30 min/3× /wk. 5-min warm up and 5-min cool down	Clinical symptoms and biochemical parameters	0	Significant reduction in ovarian volume and testosterone, and DHEA-S and SHBG levels
Nybacka et al^[17]^	4 mo	Total daily caloric intake reduced by ≥600 kcal/d, while maintaining well-balanced diet endurance, aerobic, and/or weight training.	Ovarian function	24.5%	BMI was reduced more. The menstrual pattern improved, and ovulation confirmed
De Frène et al^[24]^	24 wk	Thirty-minutes consultations calorie restriction of 450–850 kcal/d, counting the number of steps per day by means of a pedometer	BMI Quality of life	25.8%	Overall increase in HRQoL Decrease of 5% in BMI
Turan et al^[35]^	8 wk	Structured exercise program each session 50 to 60 min, 3 times/wk	Menstrual cycle Total and free testosterone BMI	12.5%	The mean menstrual cycle interval decreased significantly in the training group (*P* = .04)
Almenning et al^[37]^	10 wk	Three weekly exercise sessions. At least 1 session/wk was supervised by an exercise physiologist with normal diet	Testosterone, SHBG, and FAI BMI	19.4%	Improvement of reproductive function
Stefanaki et al^[40]^	8 wk	Mindfulness stress management program, of a 30-min audio CD of directed mindfulness and diaphragmatic breathing exercises daily, before bedtime	BMI Quality of life: PCOSQ	35%	DASS 21 depression (*P* = .011) and stress subscales (*P* = .025)
Ramos et al^[27]^	16 wk	Three series of 10 repetitions of each exercise for a period of 2 wk or 6 sessions of adaptation of 50 min	Testosterone level The SF-36 multidimensional questionnaire for quality of life	24.2%	Significantly lower level of testosterone, no significant changes in BMI, significant improvement in the score for functional capacity
Vizza et al^[33]^	12 wk	Two supervised and 2 unsupervised (home-based) training sessions per week for 12 wk	Psychological Reproductive function and quality of life (PCOSQ)	33.3%	No significant different in menstrual cycle between groups (*P* = .503). Significantly improvement 3 of 5 PCOSQ domain scores compared with SF-36 and DASS 21
Deepthi et al^[19]^	8 wk	Walking and running on treadmill 3 sessions/wk, each lasting 45 min	Quality of life, reducing BMI and number of follicles	0%	Improved the quality of life by reducing the number of follicles and BMI
Vasheghani-Farahani et al^[38]^	12 wk	Walk with a medium intensity for 30 min/d, 5 d/wk	BMI Reproductive function	25%	4 women got pregnant and reduction of BMI not statistically significant
Mani et al^[28]^	12 mo	Seven hours of interactive discussions, including patient and professional story, diet and physical activity, balancing life with PCOS, and self-management	QoL PCOSQ and SF-12 Biochemical (testosterone) and BMI	38%	Improved their QoL in 3 dimensions of PCOSQ and the SF-12 questionnaire; mean difference 5.79 (95% CI, 1.74–9.84; *P* = .006)
Costa et al^[20]^	16 wk	Progressive aerobic exercise for 40 min/d, 3 times/wk (≈150 min/wk)	Quality of life	0	HRQoL: physical functioning and mental health (*P* < .05)
Oberg et al^[18]^	4 mo	Group meetings 3× a month about; weight control, mindfulness, physical activity, and diet	Menstrual regularity and ovulation and pregnancy rates	16%	Significant BMI reduction (*P* = .002), improved menstrual regularity, no difference in ovulation rate
Cochrane et al^[23]^	12 wk	Aerobic, aqua aerobic, and gym Sessions at least 2, 1 h sessions of supervised exercise classes	BMI Menstrual regulation	0%	BMI decreased significantly (*P* < .05), improvement in menstruation pattern
Liu et al^[29]^	8–12 gestational weeks to end of pregnancy	Diet and exercise guidance by a trained dietitian; 20–25 kcal/kg of was recommended Aerobic exercises 30 min/d, 5 d/wk	Perinatal outcome Gestational weight gain	66.4%	No difference in weight gain and neonatal outcomes; premature rupture of fetal membrane, fetal distress, and preterm delivery
Benham et al^[39]^	6 mo	Continuous aerobic exercise training High-intensity interval training	Menstrual cycle, luteal phase length and numbers of ovulation events, pregnancies, and BMI	21.6%	Better vaginal lubrication and overall sexual function 5 yr after the intervention
D’souza et al^[34]^	6 mo	Counseled for healthy diet based on the BMI, brisk walk, and jogging for 30 min. Core muscle exercises, each exercise performed for 20 min/d, 5 d/wk	Menstrual irregularities, clinical feature, and quality of life	Not mentioned	A significant improvement in the waist-hip ratio, hirsutism acne (*P* value < .001) and quality of life

BMI = body mass index, BW = body weight, CD = compact disc, CI = confidence intervals, DASS 21 = Depression Anxiety Stress Scales, DHEA-S = Dehydroepiandrosterone sulfate, FAI = free androgen index, HRQoL = health-related quality of life, PCOS = polycystic ovary syndrome, PCOSQ = Polycystic Ovary Syndrome Questionnaire, QOL = quality of life, SF-36 = Short Form (36) Health Survey, SHBG = sex hormone binding globulin.

Exercise programs have also shown significant benefits, particularly structured aerobic or resistance training regimens. The efficacy of exercise alone was assessed in 11 studies.^[19,20,22,25,27,32,33,35,37–39]^ Several structured training programs were followed, such as endurance and resistance exercises with low-, high-, and medium-intensity training. Exercise, particularly structured and supervised programs, improves reproductive function by reducing ovarian volume, lowering testosterone levels, and enhancing insulin sensitivity. A 3-month structured exercise program led to a 60% normalization of menstrual cycles and significant reductions in BMI and testosterone levels. The 8-week structured exercise program significantly reduced the mean menstrual cycle interval (*P* = .04), indicating improved menstrual regularity. A 6-month high-intensity interval training program improved menstrual cycle characteristics, including luteal phase length, ovulation events, and pregnancy rates, along with enhanced BMI reduction.

Behavioral and combined interventions offer a more holistic approach, targeting both the physical and psychological aspects of PCOS. Three studies used behavioral modification programs that included mindfulness stress management and nutritional counseling.^[18,28,36]^ Behavioral interventions are highly effective in improving psychological well-being and quality of life, making them essential components of a holistic PCOS management plan. Mindfulness-based stress management programs reduced depression (*P* = .011) and stress (*P* = .025) while enhancing the overall quality of life.^[40]^ The interventions in the remaining studies (n = 6) were applied using a combined approach, including either 2 or 3 interventions.^[23,24,26,29,34,41]^ Studies integrating dietary, exercise, and behavioral components have demonstrated the most comprehensive improvements.^[23,24,26,29,34,41]^

The measured outcome was reproductive health in 16 studies, quality of life in 4 studies,^[19,20,24,36]^ and another 4 studies evaluated the lifestyle intervention on reproductive health in addition to psychological and mental health and quality of life collectively.^[27,28,33,34]^ Combined interventions address the multifactorial nature of PCOS, delivering improvements across reproductive, metabolic, and psychological domains, albeit with higher dropout rates in long-term programs.

The heterogeneity of the included studies underscores the need for individualized approaches to PCOS management. Differences in baseline participant characteristics, intervention protocols, and outcome measures suggest that a personalized strategy tailored to the unique needs of each patient may yield the best results. These findings highlight the importance of designing interventions that are both effective and adaptable to diverse presentations of PCOS.

### 3.4. Risk of bias

**Figure 2.**
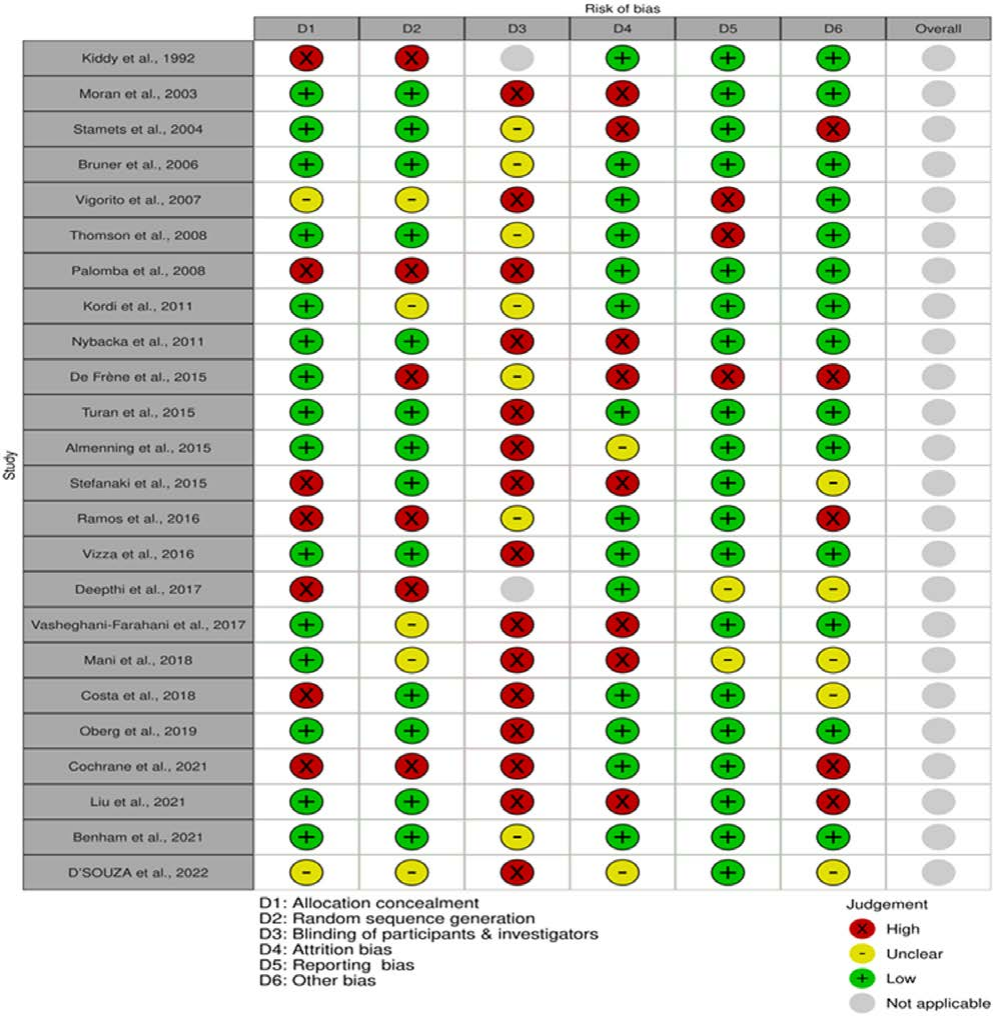
Risk of bias assessment of included studies using Cochrane Risk of Bias 2 (RoB 2) tool. This figure summarizes the assessment across 6 domains (D1–D6) for each study, where D1 represents allocation concealment, D2 represents random sequence generation, D3 represents blinding, D4 represents reporting bias, D5 represents attrition bias, and D6 represents other biases. Green circles indicate “low risk,” yellow circles indicate “some concerns,” and red circles indicate “high risk.”

All included studies were evaluated for the quality of the methodology and risk of bias based on the Cochrane collaboration tool^[42]^ that included selection bias (allocation concealment and random sequence generation), performance and detection bias, attrition and reporting bias, and any other type of bias that was not described in the above-mentioned types. The risk of bias is shown in Figure 2.^[43]^

Appropriate concealment of allocation of participants and random sequence generation were observed in 13 studies.^[17,18,26,28–33,35,37,39,41]^ Vasheghani et al^[38]^ reported that, despite the low risk of allocation concealment, the risk of random sequence generation was not clear. In contrast, it was not possible to determine from 2 trials whether adequate allocation concealment occurred, which could have led to selection bias.^[25,34]^ Investigators described that a high risk of selection bias was described by the investigators^[21,27]^ as a study design within group or without RCT, respectively. Palomba et al^[22]^ reported that the allocation of interventions was based on participant preferences. Stefanaki et al^[40]^ stated that no concealment was used within groups. Costa et al^[20]^ could not randomize all participant allocation to exercise or control groups; Cochrane et al^[23]^ stated “The subjects were not randomly assigned to groups,” while De Frène et al^[24]^ used convenience sampling.

Due to the nature of the interventions, participants and investigators, in most of the research, were not blinded as it is not easy to blind the intervention group or the caregiver to exercise, diet, or behavioral interventions, which put them at an increased risk of performance and detection bias.

Good adherence with no dropout was reported in 5 studies.^[19,20,23,25,32,41]^ The withdrawal rate was not mentioned in 1 study.^[34]^ Kiddy et al^[21]^ reported that, even though many participants missed follow-up, according to the author, their results were included in the report. Stefanaki et al^[40]^ reported a withdrawal rate of 35% in the control group compared with 0% in the intervention group. A high drop rate of >25% was observed in 8 studies.^[24,26,28,30,31,33,36,38]^ Liu et al^[29]^ despite their application of messaging app (WeChat) to reduce dropouts and improve adherence to the program, had reported a very high noncompliant rate (66%) in the intervention group according to their definition per protocol of attending at least 7 sessions of the program. Another limitation is that the study depended on the participants’ self-report in determining the compliance rate. No reason was specified for this noncompliance.

For the remaining studies, the drop rate ranged from 12% to <25%.^[17,18,22,27,35,37,39]^

Reporting bias was demonstrated in 2 studies.^[24,26]^ An increased number of missing data at different moments in time was reported by De Frène et al^[24]^. This was because of women who failed to complete the questionnaire and dropped from the trial. Thomson et al^[26]^ published no conclusive findings regarding reproductive function.

Another type of bias reported by Cochrane et al,^[23]^ was that the participants in the control group were significantly older than those in the intervention group (*P* < .05).

## 4. Discussion

This systematic review was conducted to establish the efficacy of lifestyle modifications (diet, exercise, and behavioral therapy in single or combined programs) in the management of PCOS while addressing the challenges and barriers to their implementation.

PCOS was diagnosed according to the Rotterdam criteria in most studies, which were used alone or in combination with other definitions, such as ESHRE/ASRM/NIH. The Rotterdam criteria are the most widely used categorization of PCOS worldwide according to international evidence-based recommendations, which endorse the use of Rotterdam criteria for the diagnosis of adult women with PCOS and the majority of studies and guidelines.^[44]^ Obesity and PCOS have a powerful detrimental impact on reproductive, metabolic, and psychological health, posing serious public health concerns that necessitate both prevention and treatment. Seventy-five percent of thin women and 95% of overweight women have insulin resistance.^[40]^ According to UK recommendations for the treatment of obese women with PCOS, weight management is recommended before starting ovarian stimulation therapy, preferably with a BMI of <30 kg/m^2^.^[41]^

Structured lifestyle intervention programs used in the management of PCOS, including exercise, diet, and behavioral management, either as monotherapy or in combination with 2 or 3 interventions, are considered the first-line nonpharmacological management strategy.^[45]^

Four studies in this review used dietary modifications alone to evaluate their effects on reproductive health.^[17,21,30,31]^ These studies have focused on diet composition and weight loss in obese individuals; however, studies have addressed the effects of varying diet composition on the obese PCOS population. The underlying mechanisms of improved reproductive function in overweight/obese women with PCOS in these studies appeared to involve increased insulin sensitivity. Some of these studies have emphasized the importance of balanced diet control and weight loss, even in the short term. However, moderate weight loss during long-term calorie restriction is associated with marked clinical improvements in menstrual function and fertility. An energy deficit of 30% or 500 to 750 (1200–1500) kcal/d for women could be recommended to help overweight patients lose weight. Individualized energy needs, body weight, and exercise levels should also be considered.^[46]^ Hypocaloric diets are advantageous in generating fast and significant weight loss, which plays a critical role in the amelioration of the PCOS phenotype, in addition to their role in improving insulin sensitivity and glycemic control. The efficacy of exercise alone was assessed in 10 studies included in this review.^[19,20,22,25,27,32,33,35,37–39]^ Currently, the International Androgen Excess and PCOS Society strongly recommends regular exercise of ≥150 minutes per week to maintain good body health quality, with at least 90 minutes of moderate intensity exercise (heart rate, 150 times per minute), such as playing team sports such as football or basketball or brisk walking and running.^[47]^ Regular exercise can help patients with PCOS and insulin resistance in 2 ways. First, patients with PCOS who regularly exercise can have reduced visceral fat, which subsequently leads to weight reduction. Visceral fat is more metabolically active and closely associated with insulin resistance. Second, exercise could control the activation of insulin signaling proteins in skeletal muscle, enhance muscle cell metabolism, and improve insulin sensitivity in patients with PCOS. Adults were advised to participate in muscle-strengthening activities on 2 separate days. Muscle-strengthening exercises should be minimally added to moderate-to-intense physical exercise 3 times per week.^[47,48]^ Generally, it is recommended to reduce sedentary and inactivity duration. Although different studies suggest that exercise in combination with a hypocaloric diet has a stronger ability to enhance weight loss, regular exercise or physical activity is still regarded as helpful for women with PCOS.^[49]^

Twenty-two of the studies in the current review evaluated the effectiveness of lifestyle modifications in overweight and obese women with a BMI of ≥25 kg/m^2^ and the impact of weight reduction on reproductive health and patient psychological well-being and quality of life. Similarly, several studies have shown that weight loss can improve the fundamental phenotype of PCOS, lower circulating androgen levels, and cause spontaneous resumption of menses^[15,18,23,24,28]^ and pregnancy; these findings are consistent with those of other studies.^[47,48]^ These changes have been reported, even with a relatively modest weight loss of as little as 5% of the initial body weight.^[14,18]^ Two studies in the current review evaluated the effect of exercise and behavioral lifestyle interventions, in nonoverweight and nonobese women, respectively, but using different outcomes. Turan et al^[32]^ evaluated the efficacy of exercise on reproductive health and reported a significant improvement in menstrual cycle regulation in the training group (*P* = .04). Stefanaki et al,^[40]^ who used quality of life as an outcome of endpoint measurement, found significant differences between the intervention and control groups in the depression subscales Depression Anxiety Stress Scales (*P* = .011) and stress (*P* = .025).

Three studies in this review used mindfulness stress management and nutritional counseling.^[18,28,36]^ The integration of behavioral and psychological methods, such as setting objectives, self-monitoring, cognitive restructuring, problem resolution, and prevention of relapse, in weight control strategies for women with PCOS is likely to increase the program’s effectiveness. These drugs can be used to treat women with PCOS at various times during their reproductive lives.^[47]^ Women with PCOS who participated in group training and counseling experienced improvements in weight and waist size.^[44]^

Behavioral management programs are essential treatment strategies for PCOS, and all healthcare providers should be aware of the need for counseling regarding PCOS management. Best patient counseling would result in improved overall treatment practice with the promotion of patient satisfaction. Six studies introduced a combined approach, including 2 or 3 interventions.^[16,23,24,26,34,41]^ Combined dietary, physical activity, and behavioral and weight management are first-line therapy in international evidence-based guidelines for PCOS.^[45]^ Furthermore, there is growing research on the potential benefits of including psychological and sleep interventions, as well as traditional or complementary approaches, for optimal management of PCOS.^[4]^ However, there are many challenges in the application of evidence-based lifestyle changes at different levels, irrespective of the recommended guidelines. Setting SMART (Specific Measurable, Achievable, Realistic, and Timely) goals and keeping track of one’s progress might help attain realistic lifestyle objectives. Several barriers to lifestyle management appear to be common in different healthcare systems, exposing gaps in medical knowledge on how to integrate lifestyle management.^[50]^ In general, many patients with PCOS express dissatisfaction with the advice given to them regarding lifestyle management. Insufficient communication between medical professionals creates difficulties in early diagnosis and treatment, limited utilization of allied health, a lack of follow-up and enforcement of intervention programs, and insufficient information delivery, all of which worsen patient satisfaction. Lathia et al^[39]^ in their study related the poor adoption of an active lifestyle to inadequate information given to the patient, low self-confidence in addressing the problem, long duration and type of intervention used, and short consultation time.^[44]^ The younger age of patients with PCOS appeared to be one of the factors associated with poor application of lifestyle changes in one study involved in this systematic review.^[41]^ The researcher described taking care of small children, school, and work as difficulties in adhering to the exercise program. Others reported the place of living and nature of exercise as obstacles to poor adherence to the exercise program.^[20,31]^ Stamets et al^[31]^ also stated that failure to comply with the calorie restriction or type of diet used as a result of the improper implementation of the diet by participants was a common leading cause, particularly during the first week. Benham et al^[39]^ related low exercise adherence to a lack of time, physical limitations, fear of injury, and lack of confidence. Pregnancy, illness, loss to follow-up, and personal issues, which are not specified, were additional barriers reported by 3 more studies involved in this systematic review.^[27,30,37]^ Effective promotion of adherence requires individualizing the exercise in terms of form, intensity, duration, frequency, and demands and interests.

## 5. Strengths, limitations, and future directions

This systematic review has several strengths that enhance its value in the existing literature on PCOS management. By synthesizing evidence from 24 studies involving 1373 participants, this study provides a comprehensive overview of the impact of dietary, exercise, and behavioral interventions on reproductive, metabolic, and psychological outcomes. The inclusion of diverse interventions allows for a broader understanding of how various lifestyle modifications influence multifaceted health challenges associated with PCOS. Furthermore, the use of the Cochrane Risk of Bias 2 tool ensured a rigorous evaluation of the study quality, adding credibility to the findings. Another notable strength is the emphasis on real-world applicability, as the review highlights the effectiveness of nonpharmacological strategies, which are often preferred by patients and aligned with holistic care approaches.

Despite these strengths, this review has some limitations. The substantial heterogeneity among the included studies in terms of diagnostic criteria, intervention types, and outcome measures posed challenges to data synthesis and precluded the possibility of conducting a meta-analysis. Differences in participant characteristics, such as BMI ranges and baseline reproductive health, further limited the generalizability of the findings. Moreover, the variability in intervention duration, ranging from 8 weeks to 12 months, raises questions about the sustainability and long-term impact of the reported improvements.

Adherence to interventions has emerged as another concern, with several studies reporting high dropout rates, particularly for longer and more intensive programs. This inconsistency in compliance could have introduced a bias and influenced the reliability of the results. Furthermore, while reproductive and metabolic outcomes are commonly reported, psychological and quality-of-life measures are underrepresented, leaving a critical gap in understanding the broader benefits of lifestyle interventions. The lack of standardized tools for measuring these outcomes also complicates the comparisons across studies.

Future research should address these limitations by adopting standardized diagnostic criteria for PCOS and uniform protocols for interventions and outcome assessments. Long-term studies with extended follow-up periods are crucial to understand the sustainability of lifestyle interventions and their potential to prevent complications associated with PCOS. Given the heterogeneity of the syndrome, personalized intervention strategies that consider individual differences in age, BMI, insulin resistance, and psychological well-being are essential.

Expanding the scope of the research to include psychological and quality-of-life outcomes is equally important. Interventions targeting mental health and stress management should be integrated into future studies, with a focus on evaluating these aspects using validated tools, such as the Polycystic Ovary Syndrome Questionnaire or Short Form (36) Health Survey. In addition, exploring strategies to enhance adherence, such as incorporating digital health technologies, such as mobile apps and telemedicine, could improve the feasibility and effectiveness of lifestyle interventions.

Finally, the cost-effectiveness of these interventions warrants further investigation to inform healthcare policies and resource allocation. By addressing these areas, future research can build on the strengths of this review while overcoming its limitations, ultimately advancing the management of PCOS and improving the quality of life of women affected by this complex condition.

## 6. Conclusion

This systematic review highlights the efficacy of lifestyle interventions, including dietary, exercise, and behavioral strategies, in improving reproductive health, metabolic outcomes, psychological well-being, and quality of life in women with PCOS. Despite significant heterogeneity among the included studies, consistent trends demonstrated that weight reduction and improved insulin sensitivity are key predictors of positive outcomes. Dietary interventions, particularly low-calorie and high-protein diets, are effective in improving menstrual regularity, ovulation rates, and hormonal balance, whereas structured exercise programs contribute significantly to reducing BMI and androgen levels. Behavioral therapies, though less frequently studied, are promising in addressing the psychological dimensions of PCOS, such as depression and stress, thereby supporting a more comprehensive management approach.

However, the variability in study designs, populations, interventions, and outcome measures underscores the need for standardization in future research. Long-term studies with harmonized protocols and validated tools are essential to assess the sustainability of lifestyle intervention benefits and address underexplored areas such as psychological health and adherence challenges.

This review underscores the multifactorial nature of PCOS and the necessity of adopting personalized, integrative approaches for its management. Combining dietary, exercise, and behavioral interventions offers the greatest potential to address the complex and heterogeneous manifestations of PCOS. Future research should prioritize tailoring interventions to individual patient needs, ensure adherence, and expand the focus to include cost-effectiveness and long-term quality of life outcomes. These advancements will pave the way for more effective, evidence-based, and patient-centered management strategies for PCOS.

## Acknowledgments

The authors express their sincere gratitude to all of those who supported and contributed to this study. The authors gratefully acknowledge funding from the Deanship of Graduate Studies and Scientific Research, Jazan University, Saudi Arabia, under Project Number GSSRD-24.

## Author contributions

**Conceptualization:** Amal H. Mohamed, Osama Albasheer, Manar Ahmed Ghoniem, Nagla Abdalghani, Fatma Ayish, Siddig Ibrahim Abdelwahab, Maha Murtada Abdelmageed, Ahlam Mohammed S. Hakami, Ali Hassan Khormi, Ahmed Abdallah Altraifi, Isameldin Medani, Uma Chourasia, Suhaila A. Ali, Amani Abdelmola.

**Data curation:** Amal H. Mohamed, Osama Albasheer, Manar Ahmed Ghoniem, Nagla Abdalghani, Fatma Ayish, Siddig Ibrahim Abdelwahab, Maha Murtada Abdelmageed, Ahlam Mohammed S. Hakami, Ali Hassan Khormi, Ahmed Abdallah Altraifi, Isameldin Medani, Uma Chourasia, Suhaila A. Ali, Amani Abdelmola, Anas E. Ahmed.

**Formal analysis:** Amal H. Mohamed, Osama Albasheer, Manar Ahmed Ghoniem, Nagla Abdalghani, Fatma Ayish, Siddig Ibrahim Abdelwahab, Maha Murtada Abdelmageed, Ahlam Mohammed S. Hakami, Ali Hassan Khormi, Ahmed Abdallah Altraifi, Isameldin Medani, Uma Chourasia, Suhaila A. Ali, Amani Abdelmola, Anas E. Ahmed.

**Funding acquisition:** Amal H. Mohamed, Osama Albasheer, Manar Ahmed Ghoniem, Nagla Abdalghani, Fatma Ayish, Siddig Ibrahim Abdelwahab, Maha Murtada Abdelmageed, Ahlam Mohammed S. Hakami, Ali Hassan Khormi, Ahmed Abdallah Altraifi, Isameldin Medani, Uma Chourasia, Suhaila A. Ali, Amani Abdelmola, Anas E. Ahmed.

**Investigation:** Amal H. Mohamed, Osama Albasheer, Manar Ahmed Ghoniem, Nagla Abdalghani, Fatma Ayish, Siddig Ibrahim Abdelwahab, Maha Murtada Abdelmageed, Ahlam Mohammed S. Hakami, Ali Hassan Khormi, Ahmed Abdallah Altraifi, Isameldin Medani, Uma Chourasia, Suhaila A. Ali, Amani Abdelmola, Anas E. Ahmed.

**Methodology:** Amal H. Mohamed, Osama Albasheer, Manar Ahmed Ghoniem, Nagla Abdalghani, Fatma Ayish, Siddig Ibrahim Abdelwahab, Maha Murtada Abdelmageed, Ahlam Mohammed S. Hakami, Ali Hassan Khormi, Ahmed Abdallah Altraifi, Isameldin Medani, Uma Chourasia, Suhaila A. Ali, Amani Abdelmola, Anas E. Ahmed.

**Project administration:** Amal H. Mohamed, Osama Albasheer, Manar Ahmed Ghoniem, Nagla Abdalghani, Fatma Ayish, Siddig Ibrahim Abdelwahab, Maha Murtada Abdelmageed, Ahlam Mohammed S. Hakami, Ali Hassan Khormi, Ahmed Abdallah Altraifi, Isameldin Medani, Uma Chourasia, Suhaila A. Ali, Amani Abdelmola, Anas E. Ahmed.

**Resources:** Amal H. Mohamed, Osama Albasheer, Manar Ahmed Ghoniem, Nagla Abdalghani, Fatma Ayish, Siddig Ibrahim Abdelwahab, Maha Murtada Abdelmageed, Ahlam Mohammed S. Hakami, Ali Hassan Khormi, Ahmed Abdallah Altraifi, Isameldin Medani, Uma Chourasia, Suhaila A. Ali, Amani Abdelmola, Anas E. Ahmed.

**Software:** Amal H. Mohamed, Osama Albasheer, Manar Ahmed Ghoniem, Nagla Abdalghani, Fatma Ayish, Siddig Ibrahim Abdelwahab, Maha Murtada Abdelmageed, Ahlam Mohammed S. Hakami, Ali Hassan Khormi, Ahmed Abdallah Altraifi, Isameldin Medani, Uma Chourasia, Suhaila A. Ali, Amani Abdelmola, Anas E. Ahmed.

**Supervision:** Amal H. Mohamed, Osama Albasheer, Manar Ahmed Ghoniem, Nagla Abdalghani, Fatma Ayish, Siddig Ibrahim Abdelwahab, Maha Murtada Abdelmageed, Ahlam Mohammed S. Hakami, Ali Hassan Khormi, Ahmed Abdallah Altraifi, Isameldin Medani, Uma Chourasia, Suhaila A. Ali, Amani Abdelmola, Anas E. Ahmed.

**Validation:** Amal H. Mohamed, Osama Albasheer, Manar Ahmed Ghoniem, Nagla Abdalghani, Fatma Ayish, Siddig Ibrahim Abdelwahab, Maha Murtada Abdelmageed, Ahlam Mohammed S. Hakami, Ali Hassan Khormi, Ahmed Abdallah Altraifi, Isameldin Medani, Uma Chourasia, Suhaila A. Ali, Amani Abdelmola, Anas E. Ahmed.

**Visualization:** Amal H. Mohamed, Osama Albasheer, Manar Ahmed Ghoniem, Nagla Abdalghani, Fatma Ayish, Siddig Ibrahim Abdelwahab, Maha Murtada Abdelmageed, Ahlam Mohammed S. Hakami, Ali Hassan Khormi, Ahmed Abdallah Altraifi, Isameldin Medani, Uma Chourasia, Suhaila A. Ali, Amani Abdelmola, Anas E. Ahmed.

**Writing – original draft:** Amal H. Mohamed, Osama Albasheer, Manar Ahmed Ghoniem, Nagla Abdalghani, Fatma Ayish, Siddig Ibrahim Abdelwahab, Maha Murtada Abdelmageed, Ahlam Mohammed S. Hakami, Ali Hassan Khormi, Ahmed Abdallah Altraifi, Isameldin Medani, Uma Chourasia, Suhaila A. Ali, Amani Abdelmola, Anas E. Ahmed.

**Writing – review & editing:** Amal H. Mohamed, Osama Albasheer, Manar Ahmed Ghoniem, Nagla Abdalghani, Fatma Ayish, Siddig Ibrahim Abdelwahab, Maha Murtada Abdelmageed, Ahlam Mohammed S. Hakami, Ali Hassan Khormi, Ahmed Abdallah Altraifi, Isameldin Medani, Uma Chourasia, Suhaila A. Ali, Amani Abdelmola, Anas E. Ahmed.

## References

[1] Zehravi M Maqbool M Ara I. Polycystic ovary syndrome and reproductive health of women: a curious association. Int J Adolesc Med Health. 2021;33:333–7. 33878255 10.1515/ijamh-2021-0031

[2] Ding H Zhang J Zhang F. Resistance to the insulin and elevated level of androgen: a major cause of polycystic ovary syndrome. Front Endocrinol. 2021;12:741764. 10.3389/fendo.2021.741764 PMC8564180 34745009

[3] Damone AL Joham AE Loxton D Earnest A Teede HJ Moran LJ. Depression, anxiety and perceived stress in women with and without PCOS: a community-based study. Psychol Med. 2019;49:1510–20. 30131078 10.1017/S0033291718002076

[4] Cowan S Lim S Alycia C. Lifestyle management in polycystic ovary syndrome–beyond diet and physical activity. BMC Endocr Disord. 2023;23:14. 36647089 10.1186/s12902-022-01208-y PMC9841505

[5] Yazici H Taskin MI Guney G Hismiogullari AA Arslan E Tulaci KG. The novel relationship between polycystic ovary syndrome and temporomandibular joint disorders. J Stomatol Oral Maxillofac Surg. 2021;122:544–8. 33161171 10.1016/j.jormas.2020.10.008

[6] Guney G Taşkin MI Sener N. The role of ERK-1 and ERK-2 gene polymorphisms in PCOS pathogenesis. Reprod Biol Endocrinol. 2022;20:95. 35768803 10.1186/s12958-022-00967-6 PMC9241270

[7] Camili FE Akis M Adali E. Oncostatin M is related to polycystic ovary syndrome-case control study. Biomedicines. 2024;12:355. 38397957 10.3390/biomedicines12020355 PMC10886802

[8] Parker J O’brien C Hawrelak J Gersh FL. Polycystic ovary syndrome: an evolutionary adaptation to lifestyle and the environment. Int J Environ Res Public Health. 2022;19:1336. 35162359 10.3390/ijerph19031336 PMC8835454

[9] Tokmak A Guzel AI Güney G Tasdemir U Umit C Yilmaz N. Effect of obesity on clinical parameters and pregnancy rates in women with polycystic ovary syndrome undergoing ovulation induction cycles. J Reprod Med. 2017;62:300–4. 30027724

[10] Domecq JP Prutsky G Mullan RJ. Lifestyle modification programs in polycystic ovary syndrome: systematic review and meta-analysis. J Clin Endocrinol Metab. 2013;98:4655–63. 24092832 10.1210/jc.2013-2385

[11] Adullhameed SM Abdelhafez AA ElAzab DR Alseraty WH. Effect of lifestyle changes intervention on quality of life and self-esteem of adolescent female with polycystic ovary syndrome. Int Egypt J Nurs Sci Res. 2022;2:524–33.

[12] Dietz de Loos A Jiskoot G Beerthuizen A Busschbach J Laven J. Metabolic health during a randomized controlled lifestyle intervention in women with PCOS. Eur J Endocrinol. 2022;186:53–64. 10.1530/EJE-21-0669 PMC8679850 34714771

[13] Abdolahian S Tehrani FR Amiri M. Effect of lifestyle modifications on anthropometric, clinical, and biochemical parameters in adolescent girls with polycystic ovary syndrome: a systematic review and meta-analysis. BMC Endocr Disord. 2020;20:1–17. 32429890 10.1186/s12902-020-00552-1 PMC7236342

[14] Page MJ Moher D Bossuyt PM. PRISMA 2020 explanation and elaboration: updated guidance and exemplars for reporting systematic reviews. BMJ. 2021;372:n160. 33781993 10.1136/bmj.n160 PMC8005925

[15] Sterne JA Savović J Page MJ. RoB 2: a revised tool for assessing risk of bias in randomised trials. BMJ. 2019;366. doi: 10.1136/bmj.l4898. 10.1136/bmj.l4898 31462531

[16] Yu F Liu C Sharmin S. Performance, usability, and user experience of Rayyan for systematic reviews. Proc Assoc Inf Sci Technol. 2022;59:843–4.

[17] Nybacka A Carlström K Ståhle A Nyrén S Hellström PM Hirschberg AL. Randomized comparison of the influence of dietary management and/or physical exercise on ovarian function and metabolic parameters in overweight women with polycystic ovary syndrome. Fertil Steril. 2011;96:1508–13. 21962963 10.1016/j.fertnstert.2011.09.006

[18] Oberg E Gidlöf S Jakson I Mitsell M Tollet Egnell P Hirschberg AL. Improved menstrual function in obese women with polycystic ovary syndrome after behavioural modification intervention—a randomized controlled trial. Clin Endocrinol (Oxf). 2019;90:468–78. 30565716 10.1111/cen.13919

[19] Deepthi G Sankarakumaran P Jerome A Kalirathinam D Raj NB US MR. Effect of aerobic exercise in improving the quality of life in polycystic ovarian disease. Res J Pharm Technol. 2017;10:1788–90.

[20] Costa EC Sá JCFd Stepto NK. Aerobic training improves quality of life in women with polycystic ovary syndrome. 2018. 10.1249/MSS.0000000000001579 29443823

[21] Kiddy DS Hamilton‐Fairley D Bush A. Improvement in endocrine and ovarian function during dietary treatment of obese women with polycystic ovary syndrome. Clin Endocrinol (Oxf). 1992;36:105–11. 1559293 10.1111/j.1365-2265.1992.tb02909.x

[22] Palomba S Falbo A Zullo F Orio F. Evidence-based and potential benefits of metformin in the polycystic ovary syndrome: a comprehensive review. Endocr Rev. 2009;30:1–50. 19056992 10.1210/er.2008-0030

[23] Cochrane T Tengku-Kamalden TF Davey R Omar Dev RD. Effect of exercise and weight loss in polycystic ovarian syndrome among obese women. Pertanika J Soc Sci Humanit. 2021;29:29.

[24] De Frène V Verhofstadt L Lammertyn J Stuyver I Buysse A De Sutter P. Quality of life and body mass index in overweight adult women with polycystic ovary syndrome during a lifestyle modification program. J Obstet Gynecol Neonatal Nurs. 2015;44:587–99. 10.1111/1552-6909.12739 26284937

[25] Vigorito C Giallauria F Palomba S. Beneficial effects of a three-month structured exercise training program on cardiopulmonary functional capacity in young women with polycystic ovary syndrome. J Clin Endocrinol Metab. 2007;92:1379–84. 17264174 10.1210/jc.2006-2794

[26] Thomson RL Buckley JD Noakes M Clifton PM Norman RJ Brinkworth GD. The effect of a hypocaloric diet with and without exercise training on body composition, cardiometabolic risk profile, and reproductive function in overweight and obese women with polycystic ovary syndrome. J Clin Endocrinol Metab. 2008;93:3373–80. 18583464 10.1210/jc.2008-0751

[27] Ramos FKP Da Silva Lara LA Kogure GS. Quality of life in women with polycystic ovary syndrome after a program of resistance exercise training. Rev Bras Ginecol Obstet. 2016;38:340–7. 27472811 10.1055/s-0036-1585457 PMC10374239

[28] Mani H Chudasama Y Hadjiconstantinou M. Structured education programme for women with polycystic ovary syndrome: a randomised controlled trial. Endocr Connect. 2018;7:26–35. 29133383 10.1530/EC-17-0274 PMC5744630

[29] Liu C Zhang L Zheng W. Lifestyle intervention for overweight/obese pregnant women with polycystic ovarian syndrome: lessons and challenges. Obes Facts. 2021;14:405–14. 34311460 10.1159/000514931 PMC8406241

[30] Moran LJ Deeks A Gibson-Helm M. Psychological parameters in the reproductive phenotypes of polycystic ovary syndrome. Hum Reprod. 2012;27:2082–8. 22493025 10.1093/humrep/des114

[31] Stamets K Taylor DS Kunselman A Demers LM Pelkman CL Legro RS. A randomized trial of the effects of two types of short-term hypocaloric diets on weight loss in women with polycystic ovary syndrome. Fertil Steril. 2004;81:630–7. 15037413 10.1016/j.fertnstert.2003.08.023

[32] Kordi MR Motie Z Amirsasan R. Effects of aerobic training on clinical symptoms and biochemical parameters in women with polycystic ovarian syndrome. Int J Adva Sci Res Rev. 2011;1:01–5.

[33] Vizza L Smith CA Swaraj S Agho K Cheema BS. The feasibility of progressive resistance training in women with polycystic ovary syndrome: a pilot randomized controlled trial. BMC Sports Sci Med Rehabil. 2016;8:1–12. 27175282 10.1186/s13102-016-0039-8 PMC4865007

[34] D’souza P Rodrigues DE Kaipangala RG. Effectiveness of multimodular interventions of lifestyle modification on symptoms of polycystic ovarian syndrome and quality of life among women-a quasi-experimental study. J Clin Diagn Res. 2022;16:308.

[35] Turan V Mutlu EK Solmaz U. Benefits of short-term structured exercise in non-overweight women with polycystic ovary syndrome: a prospective randomized controlled study. J Phys Ther Sci. 2015;27:2293–7. 26311969 10.1589/jpts.27.2293 PMC4540866

[36] Barber TM Hanson P Weickert MO Franks S. Obesity and polycystic ovary syndrome: implications for pathogenesis and novel management strategies. Clin Med Insights Reprod Health. 2019;13:1179558119874042. 31523137 10.1177/1179558119874042 PMC6734597

[37] Almenning I Rieber-Mohn A Lundgren KM Shetelig Løvvik T Garnæs KK Moholdt T. Effects of high intensity interval training and strength training on metabolic, cardiovascular and hormonal outcomes in women with polycystic ovary syndrome: a pilot study. PLoS One. 2015;10:e0138793. 26406234 10.1371/journal.pone.0138793 PMC4583183

[38] Vasheghani-Farahani F Khosravi S Yekta AHA. The effect of home based exercise on treatment of women with poly cystic ovary syndrome; a single-blind randomized controlled trial. Novelty Biomed. 2017;5.

[39] Benham JL Booth JE Corenblum B. Exercise training and reproductive outcomes in women with polycystic ovary syndrome: a pilot randomized controlled trial. Clin Endocrinol (Oxf). 2021;95:332–43. 33638879 10.1111/cen.14452 PMC8360032

[40] Stefanaki C Bacopoulou F Livadas S. Impact of a mindfulness stress management program on stress, anxiety, depression and quality of life in women with polycystic ovary syndrome: a randomized controlled trial. Stress. 2015;18:57–66. 25287137 10.3109/10253890.2014.974030

[41] Bruner B Chad K Chizen D. Effects of exercise and nutritional counseling in women with polycystic ovary syndrome. Appl Physiol Nutr Metab. 2006;31:384–91. 16900227 10.1139/h06-007

[42] Higgins JP Altman DG Gøtzsche PC; Cochrane Bias Methods Group. The Cochrane collaboration’s tool for assessing risk of bias in randomised trials. BMJ. 2011;343:d5928. 22008217 10.1136/bmj.d5928 PMC3196245

[43] McGuinness LA Higgins JP. Risk‐of‐bias visualization (robvis): an R package and Shiny web app for visualizing risk‐of‐bias assessments. Res Synth Methods. 2021;12:55–61. 32336025 10.1002/jrsm.1411

[44] Lathia T Joshi A Behl A. A practitioner’s toolkit for polycystic ovary syndrome counselling. Indian J Endocrinol Metab. 2022;26:17–25. 35662757 10.4103/ijem.ijem_411_21 PMC9162262

[45] Neven ACH Laven J Teede HJ Boyle JA. A summary on polycystic ovary syndrome: diagnostic criteria, prevalence, clinical manifestations, and management according to the latest international guidelines. Semin Reprod Med. 2018;36:5–12. 30189445 10.1055/s-0038-1668085

[46] Teede HJ Joham AE Paul E. Longitudinal weight gain in women identified with polycystic ovary syndrome: results of an observational study in young women. Obesity (Silver Spring). 2013;21:1526–32. 23818329 10.1002/oby.20213

[47] Teede HJ Misso ML Deeks AA; Guideline Development Groups. Assessment and management of polycystic ovary syndrome: summary of an evidence-based guideline. Med J Aust. 2011;195:S65–112. 21929505 10.5694/mja11.10915

[48] Wang S Zhang Z Liu Y. Effects of exercise intervention on the improvement of polycystic ovary syndrome. Polycystic Ovarian Syndrome: IntechOpen; 2019.

[49] Woodward A Klonizakis M Broom D. Exercise and polycystic ovary syndrome. Phys Exercise Hum Health. 2020;1228:123–36. 10.1007/978-981-15-1792-1_8 32342454

[50] Blackshaw LC Chhour I Stepto NK. Barriers and facilitators to the implementation of evidence-based lifestyle management in polycystic ovary syndrome: a narrative review. Med Sci. 2019;7:76. 10.3390/medsci7070076 PMC6681274 31252682

